# Widespread micronodular hepatic metastases of neuroendocrine tumor detected by [^68^Ga]DOTATATE PET/CT

**DOI:** 10.1016/j.radcr.2022.10.059

**Published:** 2022-11-24

**Authors:** Julia C. D'Souza, Sophia R. O'Brien, Zhaohai Yang, Amr K. El Jack, Austin R. Pantel

**Affiliations:** a Department of Radiology, Hospital of the University of Pennsylvania, 3400 Spruce St, Philadelphia, PA 19104, USA; b Division of Interventional Radiology, Hospital of the University of Pennsylvania, 3400 Spruce St, Philadelphia, PA 19104, USA; c Division of Nuclear Medicine Imaging and Therapy, Hospital of the University of Pennsylvania, 3400 Spruce St, Philadelphia, PA 19104, USA; d Department of Pathology, Hospital of the University of Pennsylvania, 3400 Spruce St, Philadelphia, PA 19104, USA; e Division of Abdominal Imaging, Hospital of the University of Pennsylvania, 3400 Spruce St, Philadelphia, PA 19104, USA

**Keywords:** Neuroendocrine tumor, DOTATATE, PET/CT, Molecular imaging, Liver, Metastases

## Abstract

Neuroendocrine tumors (NET) encompass a diverse, heterogeneous group of neoplasms that originate from the secretory cells of the neuroendocrine system. These neoplasms typically express the somatostatin receptor (SSTR), which can be targeted by molecular agents for imaging and therapy. This is particularly advantageous for imaging NETs that are indolent, slow-growing, and less well detected by [^18^F]FDG and for the detection of occult disease not easily identified by anatomic imaging. Herein, we present a case in which [^68^Ga]DOTATATE PET/CT was used to diagnose the etiology of biochemical recurrence in NET that was not apparent on MRI. The importance of understanding deviations from the normal biodistribution of the radiotracer is emphasized as key in interpreting nuclear medicine studies and establishing the diagnosis. Imaging the SSTR is of particular interest given the recent FDA approval of [^68^Cu]DOTATATE as a new and possibly more available molecular radiotracer.

## Introduction

Neuroendocrine tumors (NET) encompass a diverse, heterogeneous group of neoplasms that originate from the secretory cells of the neuroendocrine system [1], [2], [3]. These tumors predominately arise from the GI tract (often classified by embryologic location: foregut, midgut, hindgut) and bronchopulmonary systems, among other organs [2]. On a molecular level, these neoplasms express the somatostatin receptor, a class of receptors with inhibitory effects [1]. This molecular phenotype provides both imaging and therapeutic opportunities. Positron emission tomography (PET) imaging of the somatostatin receptor with [^68^Ga]DOTATATE (1,4,7,10-tetraazacyclododecane-1,4,7,10-tetraacetic acid-Tyr^3^-octreotate), and more recently [^64^Cu]DOTATATE, has become the standard-of-care in imaging these tumors, supplanting single photon emission computerized tomography (SPECT) imaging with [^111^In]Octreotide because of favorable imaging and logistical properties [4].

In this case report, we discuss a 53-year-old man with well-differentiated NET of unknown primary who presents with an increasing chromogranin A, a circulating blood biomarker of neuroendocrine tumor [2]. Initial anatomic imaging did not identify an etiology for the rising tumor marker. Molecular imaging with [^68^Ga]DOTATATE-PET revealed diffusely increased uptake throughout the liver, greater than that of the spleen and suggestive of infiltrative malignancy. This finding corresponded to a subtle perfusional abnormality of the liver parenchyma only considered suspicious for tumor in retrospect on prior MRI. In this case, repeat [^68^Ga]DOTATATE-PET/CT and identification of this altered biodistribution was key in diagnosing the etiology of progression.

## Case report

**Fig. 1:**
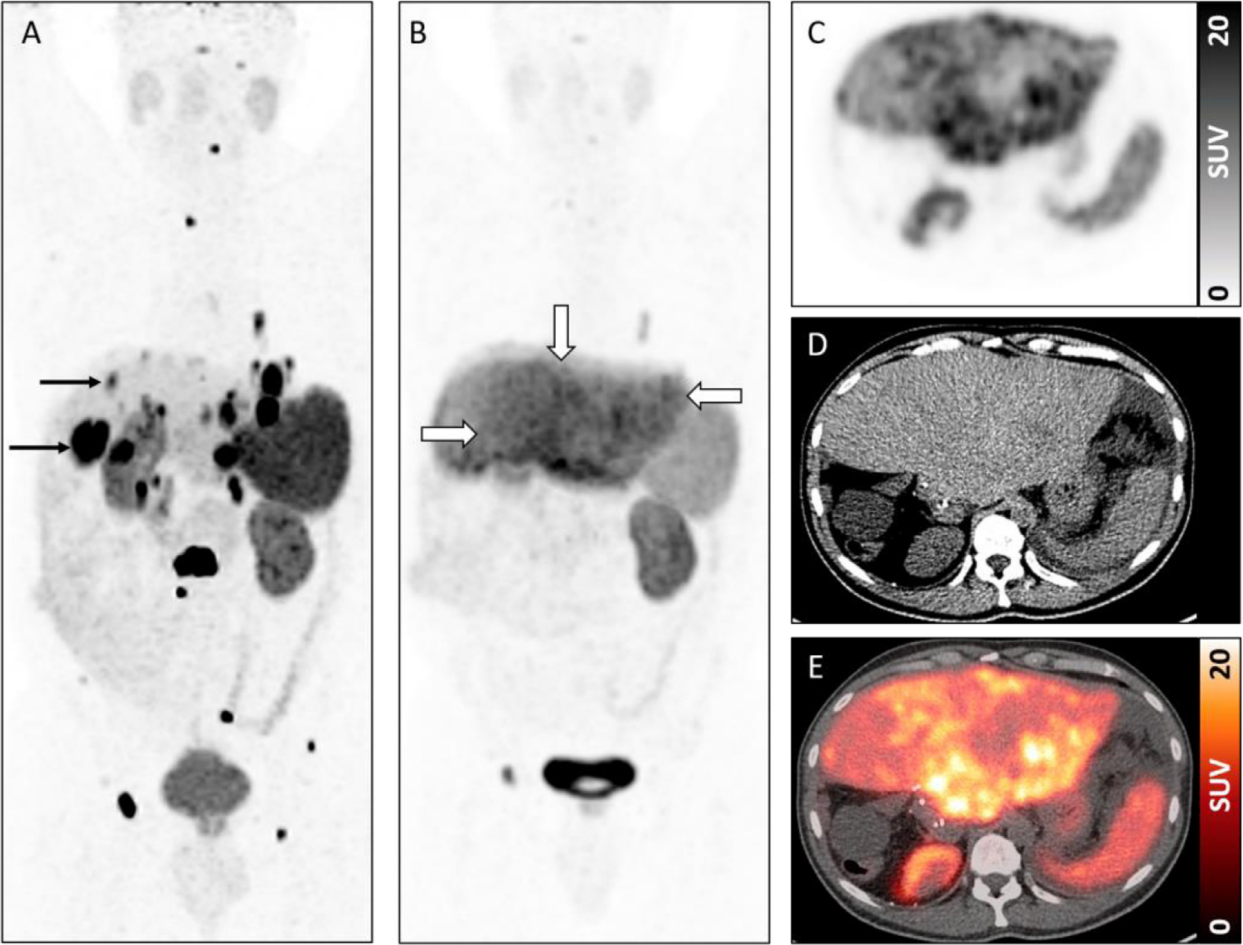
[^68^Ga]DOTATATE PET/CT from 3 years prior (A) and at the current time (B-E). Current [^68^Ga]DOTATATE PET/CT demonstrates resolution of discrete hepatic metastases seen on prior study (A), but appearance of diffusely increased uptake throughout the liver (B-E).

A 54-year-old man with well-differentiated NET metastatic to liver and bone status post multiple liver directed therapies, partial hepatic resection, and [^177^Lu]DOTATATE radionuclide therapy, presented with increasing chromogranin A (3419 ng/mL, increased from 151 ng/mL one year prior). MRI abdomen showed decreased size of known liver lesions and no new suspicious lesions. With no explanation for the rising Chromogranin A, a [^68^Ga]DOTATATE PET/CT was performed for restaging. [^68^Ga]DOTATATE PET/CT showed a decrease in number and radiotracer uptake of osseous lesions. Previously seen radiotracer-avid focal hepatic lesions on comparison ^68^Ga-DOTATATE PET/CT from 3 years prior (black arrows on coronal PET MIP in Fig. 1A) were not clearly identified on the current study above hepatic background (Fig. 1B). Instead, new heterogeneous radiotracer uptake was seen throughout the hepatic parenchyma with a SUV_mean_ of 10.8, now greater than background splenic parenchyma—representing a non-physiologic radiotracer biodistribution (white arrows in coronal PET MIP Fig. 1B) and suspicious for diffuse infiltrative disease. Additional images of the liver on the current [^68^Ga]DOTATATE PET/CT are presented (Fig. 1C axial PET, Fig. 1D axial CT, and Fig. 1E fused axial PET/CT). The prior [^68^Ga]DOTATATE PET/CT showed background liver uptake lower than spleen (liver SUV_mean_ = 4.1, Fig. 1A), reflecting the normal biodistribution.

**Fig. 2:**
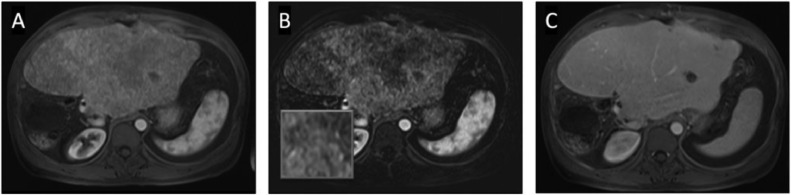
Contrast-enhanced MRI demonstrates heterogeneous arterial parenchymal enhancement (A and subtraction image B) that does not persist on delayed imaging (C).

This increased diffuse hepatic uptake of [^68^Ga]DOTATATE corresponded to a new pattern of heterogeneous hepatic parenchymal enhancement during the arterial phase of contrast when compared with MRI abdomen from 2 weeks prior (axial T1-weighted contrast-enhanced (T1W CE) MRI, Fig. 2A) with corresponding subtraction image with magnified inset (Fig. 2B) that was not apparent in later phases of enhancement (portal-venous phase axial T1W CE-MRI Fig. 2C). Though this background arterial heterogeneity had originally been considered perfusional and nonspecific, the diffuse heterogeneous uptake and altered biodistribution of [^68^Ga]DOTATATE, supported a diagnosis of diffuse infiltrative disease as the source of the patient's biochemical recurrence.

**Fig. 3:**
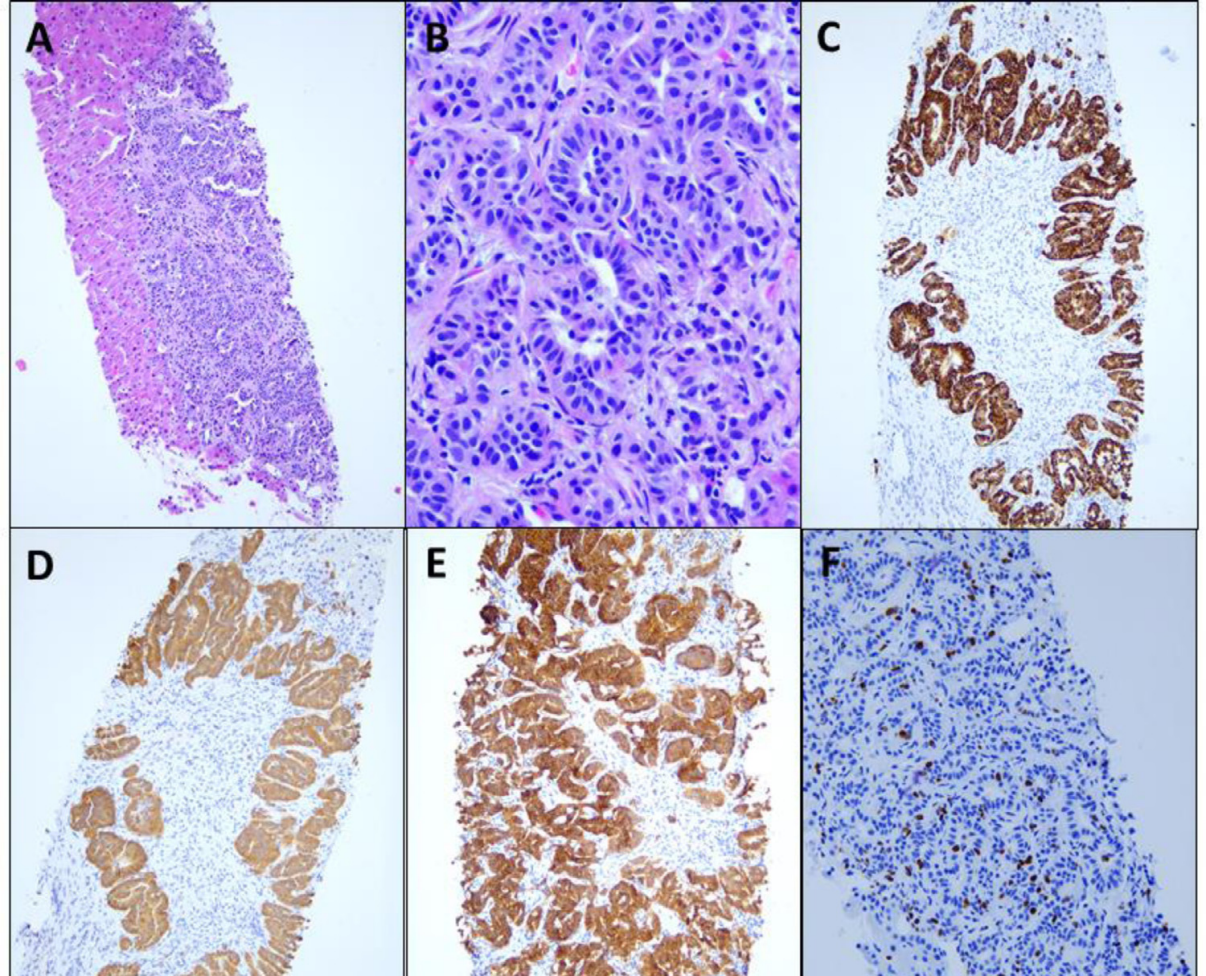
Pathology from a random liver biopsy confirmed diffuse infiltrative disease. Hematoxylin and Eosin staining demonstrated microscopic tumor foci at low power (A, original magnification ×100) and high power (B, ×400); immunohistochemistry staining was positive for chromogranin A (C, ×100) and synaptophysin (D, ×100), confirming the diagnosis of NET. Diffuse SSTR2A staining (E, ×100) confirmed the presence of the molecular target of [^68^Ga]DOTATATE, providing a biologic correlate to the PET imaging. Ki-67 staining of 8% (F, ×200) corresponds to a WHO Grade 2 malignancy.

Tissue sampling via random liver biopsy was pursued to confirm the diagnosis. This revealed multiple microscopic tumor foci (representative tumor focus at low (Fig. 3A) and high power (Fig. 3B)). The tumor cells were strongly positive for chromogranin A (Fig. 3C) and synaptophysin (Fig. 3D) on immunohistochemistry staining, confirming a diagnosis of NET. Somatostatin receptor subtype 2A (SSTR 2A) was diffusely positive in the tumor (Fig. 3E). The Ki-67 labeling index was ∼8% (Fig. 3F), corresponding to WHO Grade 2. The liver biopsy supports, as suspected on molecular imaging, a widespread miliary pattern of infiltrative disease.

## Discussion

Miliary metastatic disease in the liver represents an uncommon metastatic pattern for neuroendocrine tumor. Pancreatic, small bowel, and pulmonary NETs often metastasize to the liver, though metastases are typically focal and show intense radiotracer uptake [5,6], similar to Figure 1A. While confluent or diffuse hepatic metastases may be seen, small miliary metastases are less common [7], [8], [9].

The diffuse micronodular infiltrative disease in our case was recognized by widespread abnormally increased hepatic ^68^Ga-DOTATATE uptake that was greater than spleen—a deviation from the normal DOTATATE biodistribution. Physiologic [^68^Ga]DOTATATE uptake in the liver is less than that of the spleen [10,11], concordant with the spleen expressing higher concentrations of SSTR2, the primary target of [^68^Ga]DOTATATE [12,13]. Additionally, the high burden of infiltrative tumor may have preferentially bound the radiotracer, leading to decreased splenic uptake (SUV 11.9 versus 19.4 in 2018, Fig. 1B versus Fig. 1A, respectively), via the “sink effect” [12,14]. Thus, in our case with nonspecific MRI findings, [^68^Ga]DOTATATE played a key role in restaging occult disease, and the abnormal [^68^Ga]DOTATATE distribution was key in diagnosing the etiology of the patient's biochemical progression.

Additional roles of SSTR imaging in the management of NETs include initial staging or staging before surgery, localization of primary tumor if unknown, selection of patients for SSTR-targeted PRRT, evaluation of suspected NET in a mass not amenable to biopsy, monitoring NETs predominantly seen on SSTR imaging, and evaluation of patients with biochemical evidence of NET without known tumor or histologic diagnosis or cases of biochemical progression [15]. It must be considered whether high-grade NET may be dedifferentiated and no longer express SSTR when evaluating for presence or progression of disease. Additionally, while resolution of PET is inferior to CT or MRI, SSTR imaging is highly sensitive and specific with [^68^Ga]DOTATATE PET/CT reported as 90%-94% sensitive and 90%-92% specific [16], [17], [18].

PET imaging of radiolabeled DOTATATE has replaced [^111^In]DTPA-Octreotide (Octreoscan) SPECT imaging of the somatostatin receptor. Slow pharmacokinetics necessitating long uptake times (imaging at 4 and 24 hours), unfavorable dosimetry limiting injected activity, and high-energy emissions, combined with the known limitations of SPECT imaging, hampered the ultimate utility of the Octreoscan [18]. The first [^68^Ga]-labeled PET imaging agent targeting the somatostatin receptor, [^68^Ga]DOTATATE (the radiotracer used in this study) was approved by the FDA in 2016, followed by [^68^Ga]DOTATOC in 2019 [19]. Compared to Octreoscan, these agents benefit from lower radiation dose to the patient, less burdensome imaging protocols (image ∼1 hour after radiotracer injection), higher target affinity, and better imaging characteristics [12,20]. In a systematic review and meta-analysis, [^68^Ga]DOTATOC demonstrated superior sensitivity compared to Octreoscan [18]. The performance benefits of PET imaging of the somatostatin receptor have rendered Octreoscan obsolete in current clinical practice.

More recently in 2020, the FDA approved [^64^Cu]DOTATATE for imaging somatostatin receptor-positive malignancies. Compared to [^68^Ga], [^64^Cu] has a lower positron range, resulting in improved spatial resolution. Moreover, the longer physical half-life of [^64^Cu] (12.7 hours) compared to [^68^Ga] (68 minutes) enables centralized distribution of the radiotracer and more flexible imaging protocols (image 1 to 3 hours after injection) [21], [22], [23]. The lower positron fraction of [^64^Cu], though, may necessitate modified imaging protocols to maintain image quality. No convincing evidence has been published supporting the use of either of these PET agents over the other, and for the time being, these 2 radiotracers may be thought of as equivalent for imaging. Consideration may be given to using the same agent for imaging follow up in the same patient [21].

The benefits of imaging the somatostatin receptor with PET have become evident since the introduction SSTR molecular agents less than a decade ago. This case demonstrates the benefit of molecular imaging in NET, identifying infiltrative liver disease that appeared non-specific on MRI. With increased availability of these imaging agents and FDA approved radionuclide therapy directed at the same target ([^177^Lu]DOTATATE) [24]), radiologists should have familiarity with these agents and their role in the management and treatment of neuroendocrine tumors.

## Patient consent

This statement is to document that written consent was obtained from the patient presented in this report for publication of their de-identified images and case information for educational purposes.
